# New York University

**Published:** 1844-09

**Authors:** 


*New York University.*—We have received the annual announcement of this institution. The untiring zeal and energy displayed by the faculty to render the school under their care, the most attractive to those desiring a thorough practical education in medicine, is highly creditable. The course of lectures opens on the last Monday in October, under peculiarly favorable auspices.

